# Classification of properties and their relation to chemical bonding: Essential steps toward the inverse design of functional materials

**DOI:** 10.1126/sciadv.ade0828

**Published:** 2022-11-25

**Authors:** Carl-Friedrich Schön, Steffen van Bergerem, Christian Mattes, Aakash Yadav, Martin Grohe, Leif Kobbelt, Matthias Wuttig

**Affiliations:** ^1^ I. Institute of Physics, Physics of Novel Materials, RWTH Aachen University, 52056 Aachen, Germany.; ^2^ Chair of Computer Science 7–Logic and Theory of Discrete Systems, RWTH Aachen University, 52074 Aachen, Germany.; ^3^ Visual Computing Institute, RWTH Aachen University, 52074 Aachen, Germany.; ^4^ Ulsan National Institute of Science and Technology (UNIST), 50 UNIST-gil, Eonyang-eup, Ulju-gun, Ulsan, South Korea.; ^5^ Jülich-Aachen Research Alliance (JARA FIT and JARA HPC), RWTH Aachen University, 52056 Aachen, Germany.; ^6^ PGI 10 (Green IT), Forschungszentrum Jülich GmbH, 52428 Jülich, Germany.

## Abstract

To design advanced functional materials, different concepts are currently pursued, including machine learning and high-throughput calculations. Here, a different approach is presented, which uses the innate structure of the multidimensional property space. Clustering algorithms confirm the intricate structure of property space and relate the different property classes to different chemical bonding mechanisms. For the inorganic compounds studied here, four different property classes are identified and related to ionic, metallic, covalent, and recently identified metavalent bonding. These different bonding mechanisms can be quantified by two quantum chemical bonding descriptors, the number of electrons transferred and the number of electrons shared between adjacent atoms. Hence, we can link these bonding descriptors to the corresponding property portfolio, turning bonding descriptors into property predictors. The close relationship between material properties and quantum chemical bonding descriptors can be used for an inverse material design, identifying particularly promising materials based on a set of target functionalities.

## Teaser

Classification of properties is an essential step toward the inverse design of materials.

## INTRODUCTION

Tailoring the properties of solids is paramount to enhance the application potential of advanced functional materials. This should help, for example, to improve solar absorbers, thermoelectrics, or ionic conductors, which promise to play a prominent role in a sustainable future. Many relevant characteristics such as the optical absorption of a photovoltaic material or the relevant effective masses of thermoelectrics can, in principle, be derived from the solution of the Schrödinger equation. However, solving the Schrödinger equation with suitable precision for anything but simple systems will always be a daunting task, which can only be accomplished with adequate approximations. Density functional theory (DFT) provides a feasible strategy to determine a crucial ground state property, the total energy of the system (*1*). However, to accomplish this objective, an adequate functional is required, which is difficult to determine. Instead, many different approximations have been developed (*2*). Recently, it has even been proposed to use machine learning to develop more accurate DFT functionals (*3*). Nevertheless, for each ground state property, a (different) functional of the electron density is needed. Even if these will become available, DFT calculations are essentially a one-way street, as they determine properties for a given solid.

**Fig. 1.**
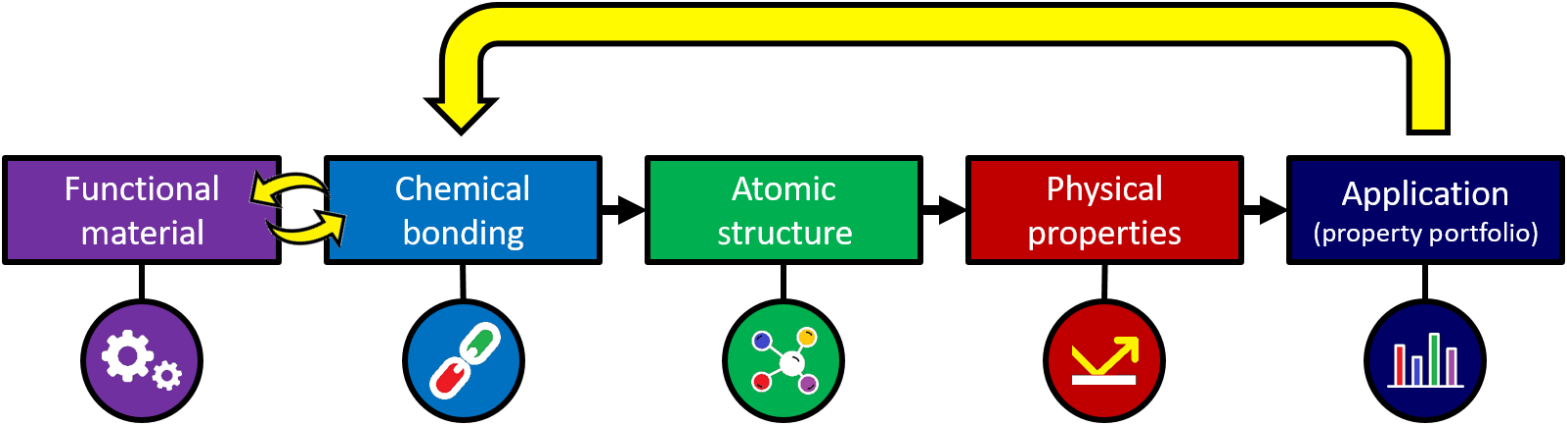
The inverse material design challenge. The properties of solids depend on the arrangement of its constituting atoms, which influence its electronic structure. This characteristic arrangement of atoms is shaped by the chemical bonding mechanism present. The property portfolio of a functional material, on the other hand, defines its application potential. It should therefore be possible to revert this relation and infer from a material’s property portfolio which bonding mechanism is present, i.e., realize inverse material design (yellow arrow). Last, one can identify suitable materials that are characterized by the corresponding quantum chemical bonding descriptors.

However, the solution of the inverse problem is what we are really looking for (*4*–*9*). For applications, a portfolio of properties is required to realize the desired system performance. For thermoelectrics, for example, a high Seebeck coefficient and a large electrical conductivity in conjunction with a small thermal conductivity are needed, as summarized in the figure of merit (*10*). Photovoltaic materials for solar absorbers require a bandgap of around 1.2 eV (*11*), a strong optical absorption, and a sufficient mobility of the charge carriers, which is facilitated by charge carriers with small effective masses. One hence needs to wonder which materials have a particularly good property portfolio for thermoelectric or photovoltaic applications (*12*). In recent years, substantial efforts have been devoted in building searchable databases (*13*, *14*) that contain vast information regarding the properties of many solids. However, identifying uncharted solids, i.e., compounds that have not yet been synthesized, is generally hard to accomplish using databases and requires a different approach. To master this challenging task, a different strategy should be beneficial. Starting from the desired property portfolio, we can ponder which material is best suited to fulfill them. Here, we are presenting a strategy on how to realize this material identification and describing several relevant steps of its implementation (see Fig. 1).

In particular, two facets of the inverse design challenge for advanced functional materials will be in focus. At first, the characteristics of property space of advanced functional materials will be explored. Properties such as the electrical conductivity, effective masses of the charge carriers, the bandgap, or, alternatively, the optical dielectric constant ε_∞_ are important for a number of applications. One can ponder whether this multidimensional property space has structure, i.e., if hidden (or even obvious) parameter correlations exist. Very high electrical conductivities at room temperature, i.e., conductivities above 10^5^ S cm^−1^, for example, cannot be reached in materials with a large bandgap but instead can be easily reached in good metals. However, there might be more intricate property correlations. In particular, it seems likely that the pronounced differences of material properties will lead to a clear separation of materials into different classes. Hence, in a first step, we will use clustering algorithms to distinguish different materials classes. It will be shown that several distinct classes can be identified, which differ considerably in their property portfolio. These different property combinations will be shown to be related to dissimilar bonding mechanisms, as can be quantified by differences in their quantum chemical bonding descriptors, which have been derived from the quantum theory of atoms in molecules (QTAIM), developed by Bader (*15*). These chemical bonding descriptors are thus apparently successful property predictors, which can be used to map material properties. The close relationship between material properties and quantum-chemical bonding descriptors can be used to identify materials, even hitherto unknown compounds, which should have tailored properties.

Finding hidden information such as property correlations within a dataset is the core topic of data science. We will hence leverage data analysis methods to classify materials according to their properties. As this algorithmic approach does not depend on any underlying model of the solid and is purely data driven (evidence driven), structures unveiled by this ansatz are a priori objective indicators unbiased by any form of model assumption. Since we want to relate material properties to chemical bonding, we need to focus on rather simple solids, where the intrinsic properties of the material can be related to a single type of bond in the system. In the concluding section of the manuscript, the extension of this approach to more complex solids is discussed.

We will begin by explaining the algorithm used, e.g., which methods were used to conduct the classification. This is followed by the presentation and discussion of the clustering results. Next, the properties used for the clustering are analyzed with respect to their significance as descriptors to distinguish between different classes. Subsequently, we will analyze property correlations and thus the structure and metric of the multidimensional property space. A technique to represent the clustering visually is presented thereafter, which yields insights into the strengths and weaknesses of the algorithm used. This visualization is also used to sketch the evolution of the clustering results for different target numbers of clusters.

Last, we will relate the property portfolio to quantitative chemical bonding descriptors. By unraveling the underlying connection between chemical bonding and physical properties, we aim to identify an alternative path to design advanced functional materials. It will be shown that the concept of chemical bonding, originally introduced as a heuristic model to explain chemistry in a quantitative way about a century ago, can become a useful tool for the design of functional materials if based on adequate quantum chemical bonding descriptors.

## RESULTS

### Materials and properties used for clustering

As mentioned in the Introduction, a characteristic property set has to be chosen (see Table 1). Including more properties promises to yield more spatially separated clustering results. However, the limited availability of the property in question for each material in the database has to be taken into consideration. As a compromise between the number of materials and the number of properties considered, the following set of properties has been chosen: (i) the electrical conductivity σ, (ii) Born effective charge Z*, (iii) effective coordination number (ECoN), (iv) bandgap *E*_G_, (v) melting point *T*_M_, (vi) density ρ, and (vii) atomic density ρ_A_. We have selected these parameters since they are easily accessible in databases (from experiment or simulation) and are well defined for all classes of solids from ionic to covalent and metallic compounds. Furthermore, they are also relevant for applications (with the exception of the ECoN and the atomic density ρ_A_). In the “From property space classification to material design” section, the properties of electrons transferred (ET) and electrons shared (ES) are introduced. It Is important to stress that these properties (ES and ET) were not used for clustering, only the seven properties listed.

**Table 1.** Different types of chemical bonds and their characteristic properties. No set of properties (of any bond type) is a linear combination of the other bond types. This provides strong evidence that these four bond types are indeed unique and not a combination or mixture of the other bonds. bcc, body-centered cubic; hcp, hexagonal close-packed; fcc, face-centered cubic.

	**Ionic (e.g., NaCl)**	**Covalent (e.g., Si, GaAs)**	**Metavalent (e.g., GeTe)**	**Metallic (e.g., Cu and NiAl)**
Electronic conductivity σ	Very low (<10^−8^ S cm^−1^)	Low moderate (10^−8^–10^2^ S cm^−1^)	Moderate (10^1^–10^4^ S cm^−1^)	High (>10^5^ S cm^−1^)
Number of nearest neighbors	4 (ZnS), 6 (NaCl), and 8 (CsCl)	8-N rule typically satisfied	8-N rule not satisfied	8 (bcc) and 12 (hcp/fcc)
Optical dielectric constant ε_∞_	Low (≈2–3)	Moderate (≈5–15)	High (>15)	—
Born effective charges Z*	Low (1–2)	Moderate (2–3)	High (4–6)	Vanishes (0)
Grüneisen parameter γ_TO_	Moderate (2–3)	Low (0–2)	High (>3)	Low (0–2)

The database of materials used for clustering is mainly taken from (*16*), supplemented by additional compounds. These materials are ideal crystals; hence, the effects of defects, which can be dominant for selected groups of materials and specific properties, are not considered within this study. The complete table of materials is presented in Table 2. As the conductivity σ varies by more than 20 orders of magnitude over the range of materials included, the logarithm of the conductivity is used. Furthermore, the Born effective charge Z* is divided by the nominal oxidation state to calculate the excess Born effective charge Z*_+_. For an ordinary ionic bond, Z* approaches the formal oxidation state, and the excess Born effective charge Z*_+_ is thus close to 1. Hence, using the excess Born effective charge, the deviation from the properties of ordinary ionic bonds is rated.

**Table 2.** Comparison of the expert-based classification with the results of the clustering algorithm. The rows denote the clusters found by the algorithm. These clusters are named after the most common bonding type within this cluster. The traditional (expert) classification is denoted by superscript, if it differs from the result of the clustering algorithm. Hence, GaN^cov.^ in the top row (cluster 1: ionic) means that the algorithm classifies this material as being ionic, while the criteria summarized in Table 1 imply it to be covalent. Blends of materials/alloys are donated by colons. The stable phases were used for most compounds; metastable structures are indicated in brackets.

**Cluster 1 (ionic)**	AgSbS2^cov.^, AlN, AlP, BaO, BaS, BaSe, BaTe, BeO, BeS, BeSe, BeTe, BN, C-Diamond^cov.^, CaO, CaS, CaSe, CaTe, CsBr, CsCl, CsF, CsF ($\mathrm{Pm}\bar{3}m$), CsI, GaN^cov.^, GeSe^cov.^, HgS ($F\bar{4}3m$)^cov.^, KBr, KCl, KF, KI, MgO, MgS, MgSe, MgTe, NaCl, NaBr, NaF, NiO^cov.^, PbO^cov.^, RbBr, RbBr ($\mathrm{Pm}\bar{3}m$), RbCl, RbI, Sb_2_S_3_^cov.^, Sb_2_Se_3_^cov.^, SnO^cov.^, SnS^cov.^, SrO, SrS, SrSe, SrTe, ZnO^cov.^, ZnS^cov.^, ZnSe^cov.^, RbBr:RbI, NaBr:NaCl, NaCl:KCl, CaS:CaSe, CsCl:CsBr, and CsBr:CsI
**Cluster 2 (covalent)**	AlBi, AlSb, CdS, CdSe, CdTe, GaP, GaAs, GaSb, GaSe, Ge, HgSe, HgTe, InAs, InP, InSb, Si, SnSe (*Pnma*), ZnTe, CdS:CdSe, CdTe:HgTe, HgSe:HgTe, InP:GaP, InAs:GaAs, InP:InAs, GaP:GaAs, InP:GaP:InAs:GaAs, InP:GaP:InSb:GaSb, and InAs:GaAs:InSb:GaSb
**Cluster 3 (metavalent)**	AgSbSe_2_, AgBiSe_2_, AgBiTe_2_, AgSbTe_2_, As_2_Te_3_ ($R\bar{3}m$), Bi_2_Se_3_, Bi_2_Te_3_, GeTe, PbS, PbSe, PbTe, Sb_2_Te_3_, SnTe, PbS: PbSe, PbTe:PbSe, PbTe:SnTe, GeTe:SnTe, Sb_2_Te_3_:Bi_2_Te_3_, Bi_2_Se_3_:Bi_2_Te_3_, PbTe:AgSbTe_2_, GeTe:AgSbTe_2_, AgSbTe_2_:AgBiTe_2_, GeTe:Sb_2_Te_3_, SnTe:Sb_2_Te_3_, PbTe:Sb_2_Te_3_, and PbTe:Bi_2_Te_3_
**Cluster 4 (metallic)**	Ag, AgSnSe_2_, AgSnTe_2_, Al, Au, Ca, Ca ($\text{Im}\bar{3}m$), Cu, CuAu, Hf, K, Li, Mg, Na, Nb, Ni, NiPt, Pb, Pd, Sc, Sr, Ta, Y, Zr, AlAu, AlCu, AlPd, AlPt, Co, Cu, CuZr, Fe, Ga, GaPd, GaPt, In, In_3_SbTe_2_, Ir, La, Mn, Mo, NbC, NbN, Ni, NiAl, Pt, Re, Rh, TiO, Zn, Ni:Pd, Ni:Cu, Sr:Ca, Hf:Zr, Au:Co, Co:Ni, and Ta:Nb

The total number of unique data points used amounts to approximately 130 different materials. To augment the density of data points, miscible materials (as confirmed by their phase diagram) were selected, and their properties were interpolated. This procedure creates intermediate data points and extends the database to about 330 compounds. The linear interpolation is motivated by Vegard’s law (*17*). Each material is treated as a point in a seven-dimensional (7D) property space, characterized by σ, Z*, ECoN, *E*_G_, *T*_M_, ρ, and ρ_A_.

### Clustering algorithm and its results

The whole set of data points is analyzed by leveraging (a variant of) the expectation maximization algorithm (EM algorithm) (*18*) to fit a Gaussian mixture model (GMM). By varying the number of modes, the number of clusters formed is controlled. Hence, with this approach, the coarse-grained structure of property space is explored and used for material clustering. This material clustering is subsequently related to distinct material types. As will be shown in the subsequent paragraphs, the material types identified by the clustering seem to correlate to different types of chemical bonding. Textbooks on solid-state physics and material science typically present and discuss five different types of bonding: covalent, ionic, and metallic bonding, as well as van der Waals and hydrogen bonding. The database does not contain materials that use van der Waals or hydrogen bonding (such as ice). Hence, the clustering is expected to yield the best result for three clusters: one cluster for ionic, one for covalent, and one for metallic bonding. This hypothesis can be verified by computing the average log likelihood over all samples as a measure for the clustering quality. The average log likelihood is then plotted as a function of the number of clusters, and an “elbow” analysis is conducted to locate the optimum that pareto-optimizes clustering quality and regression efficiency (see fig. S2).

Besides the clustering by the EM algorithm with minimal human interference, a second approach has been used to classify materials for comparison. This method focuses on a somewhat different set of only five material properties (see Table 1). These properties have been specifically selected to distinguish different bonding mechanisms based on criteria devised by material scientists (*19*). The first quantity is the electrical conductivity at room temperature, a measure of the electronic structure of the material. With this quantity, it is relatively easy to separate metals, which are characterized by room-temperature electrical conductivities above about 10^5^ S cm^−1^. The atomic arrangement as described by the ECoN (*20*), a distance-weighted measure of the number of nearest neighbors, has been used as a quantitative identifier of the structure. This quantity helps to identify covalent bonds that often have a number of nearest neighbors, which correspond to 8-*N*, where *N* is the average number of valence electrons (8-*N* rule) (*21*, *22*). The third quantity is the high-frequency dielectric constant ε_∞_, an optical identifier. The fourth quantity is the Born effective charge Z*, a measure of the chemical bond polarizability. Last, the Grüneisen parameter for transverse optical modes γ_TO_ has been used as a measure of the lattice anharmonicity. By using these five parameters (or a sufficient subset), it is rather straightforward to separate metallic, ionic, and covalent bonding. However, with these quantities, compelling evidence for another distinct bonding mechanism besides metallic, ionic, and covalent bonding has been found. This bonding mechanism has been coined metavalent bonding (MVB) (*16*, *19*, *23*–*25*). It is characterized by a rather soft, i.e., anharmonic, lattice, evidenced by large values of the Grüneisen parameter γ_TO_ and an electrical conductivity that approaches the room-temperature conductivity of metals but is about one to two orders of magnitude lower. Furthermore, metavalent solids have an ECoN larger than that expected for ordinary covalent bonds (8-N rule). In addition, metavalent solids retain large optical dielectric constants ε_∞_, indicative of a special orbital arrangement. Last, these materials have high values of the Born effective charge, a measure of the chemical bond polarizability Z*.

Evidence for a distinct previously unidentified bonding mechanism should be taken with considerable caution, given the fact that the established bonding mechanisms of metallic, ionic, and covalent bonding have been identified decades ago. They are frequently considered as hypothetical, i.e., prototypical corner cases, while “real” bonds in solids might well be a mixture of different bonding mechanisms. NaCl, for example, is usually the prime example of ionic bonding, held together solely by electron transfer and the resulting electrostatic forces; however, there is still a miniscule amount of electron sharing present, which is the mechanism driving covalent bonding. Similar statements could be made about the other bonding types. Hence, it is interesting to ask whether a purely data-driven analysis of material properties, which even works with a different set of properties, also finds evidence for the same four classes of materials. Note, however, that it is not the main purpose of this paper to reveal an unknown chemical bonding type, i.e., MVB. The classification of MVB as a distinct bonding mechanism has already been achieved in another recent publication, which uses quantum chemical bonding descriptors based on the Hirshfeld analysis for this task (*26*). Our prime goal is the classification of material properties to prepare an alternative route to the inverse design of materials, particularly those that exhibit quite peculiar and interesting material characteristics, in view of technological applications.

**Fig. 2.**
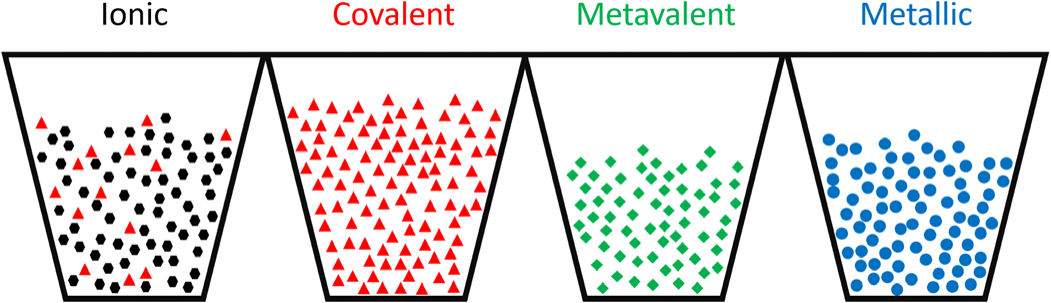
Overview of the clustering results for four clusters. Each container represents a cluster found by the algorithm, while the colors and symbols indicate the bonding type of the corresponding compound by the criteria of Table 1. Black hexagons, ionic; red triangles, covalent; green diamonds, metavalent; blue circles, metallic.

A comparison of the classification using the two different approaches is presented in Table 2 and illustrated in Fig. 2. The four different rows in Table 2 list the clustering according to the analysis using the purely data-driven EM approach. The superscript of each material instead characterizes the bond-type classification according to the human expert-based classification summarized in Table 1, in case it differs from the EM classification.

It is notable that the ionic, metavalent, and metallic clusters all respectively include every compound labeled the same way as done by the expert classification. While the ionic cluster has perfect recall (i.e., the proportion of correct compounds being retrieved ➔ all ionic compounds were put in this cluster) but has imperfect precision of about 82% (i.e., the proportion of retrieved items being correct ➔ not all compounds in this cluster are ionic), the covalent cluster has an imperfect recall of about 87% but features perfect precision.

This is also illustrated in Fig. 2, which shows the classification in different groups as cups, which contain colored symbols. The color and symbol type correspond to the expert-based classification using the criteria listed in Table 1, while the different containers describe the clustering by the EM approach.

Obviously, there is no problem in identifying a material as a metal. While the materials in our database classified as metals according to the criteria in Table 1 differ considerably in terms of density, melting point, or ECoN, there seems to be sufficient property coherence to characterize all of these materials including not only Al, Ag, Au, Ca, Cu, Hf, Mg, Sr, W, and Zn but also compounds such as CuAu and NiAl as metals. Even chiral metals such as AlPd, AlPt, GaPd, and GaPt are correctly classified, although they have a smaller electrical conductivity at room temperature and a smaller ECoN than good metals (Ag, Au, and Cu). The clustering algorithm also correctly identifies compounds that have been characterized as ionic according to the criteria of Table 1, including materials such as alkali halides (NaCl, CsCl, CsI, and RbBr) and several earth alkali oxides and chalcogenides such as MgO, MgS, MgSe, and MgTe.

The most interesting observation is presumably the finding that the EM algorithm also identifies a fourth class, besides ionic, metallic, and covalent compounds. In the database used, the following compounds are identified as metavalent: AgSbTe_2_, AgBiSe_2_, AgBiTe_2_, AgSbTe_2_, As_2_Te_3_ $\left(R\bar{3}m\right)$, Bi_2_Se_3_ $\left(R\bar{3}m\right)$, Bi_2_Te_3_, GeTe (*R*3*m*), PbS, PbSe, PbTe, Sb_2_Te_3_ $\left(R\bar{3}m\right)$, and SnTe. This list contains a number of rather peculiar materials that are presently used as thermoelectrics [PbTe, PbSe, AgBiSe_2_, AgBiTe_2_, As_2_Te_3_ (*R*3*m*), SnTe, Bi_2_Se_3_ $\left(R\bar{3}m\right)$, and Bi_2_Te_3_] (*25*), as phase change materials [GeTe, Sb_2_Te_3_ $\left(R\bar{3}m\right)$, and AgSbTe_2_] (*27*), and as topological insulators [Sb_2_Te_3_, Bi_2_Te_3_, Bi_2_Se_3_, SnTe, and PbTe] (*28*). Both classification approaches draw the same borders between MVB and covalent bonding and between MVB and metallic bonding. This finding is a compelling indicator that MVB is a distinct bonding mechanism in solids besides ionic, metallic, and covalent bonding.

Before further conclusions are drawn from this classification, some comments are in place concerning the weaknesses of the present clustering. There are difficulties concerning the distinction between ionic and covalent bonding as can be seen from the disagreement of the two algorithms in assigning, e.g., GaN, GeSe, SnS, and PbO. Pure covalent and pure ionic bonding are apparently two limiting cases, yet many solids use a bonding mechanism that encompasses elements of ionic and covalent bonding, best summarized by the term iono-covalent bonding. This impedes defining a clear boundary between ionic and covalent bonding and raises the question on how quantitative criteria could be developed that define a clear border between materials dominated by charge transfer (ionic bonding) and those governed by electron pair formation (covalent bonding). However, there is one clustering error by the EM algorithm that is not related to the diffuse nature of the boundary between ionic and covalent bonding. Diamond, which uses covalent bonding, is characterized by the algorithm as an ionic material. This is a clear outlier. One feature that sets diamond apart from (most of) the other covalent compounds is that it is monoatomic, which results in a vanishing Born effective charge by symmetry. However, the same holds true for Ge and Si, which are classified correctly. Hence, we suspect that the relatively large bandgap of diamond in conjunction with the monoatomic characteristics and the small number of covalent compounds with similar properties (large bandgap, high melting temperature, etc.) could be responsible for the misclassification. To understand the clustering better, the distribution function for different properties is shown next.

## DISCUSSION

### Structure of the property space

**Fig. 3.**
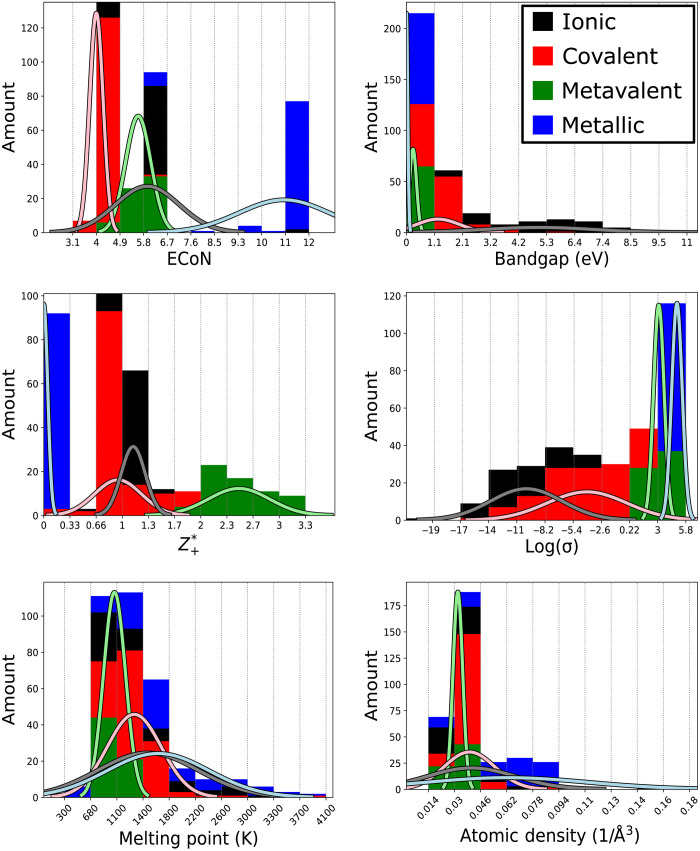
Property distribution within the (expert-) assigned bonding mechanisms. Metals are indicated in blue, covalent materials in red, metavalently bonded systems in green, and ionic compounds in black. The plots illustrate that no property by itself is sufficient to separate between all bonding types. However, some properties only exist within a relatively narrow window for a specific bonding type.

In Fig. 3, the distribution histograms for the different properties are shown. Each property spectrum is subdivided into 10 bins each, with the minimum and maximum value indicated on the *x* axes. For each property, the number of compounds in each bin is counted and represented by the length of the bar within the bin, while the color of the bar represents the bonding type. Furthermore, a Gaussian is fitted to the property distribution and plotted in the same graph.

There are certain properties that have very little, if any, predictive power concerning the prevalent bonding mechanism. This holds in particular for not only the melting temperature but also the atomic density and the mass density. The classes of ionic, covalent, and metallic compounds each span a wide range of values. The melting temperatures, for example, range between 853 and 3643 K for ionic compounds, 302 and 3873 K for metallic compounds, and 794 and 4100 K for covalent compounds, respectively. Only metavalent solids reveal a smaller range of melting temperatures spanning from 828 to 1387 K. A similar conclusion can be drawn for the atomic densities. Again, wide distributions are found for ionic, metallic, and covalent compounds, ranging from 0.017 and 0.168 1/Å^3^ for ionic compounds, 0.014 and 0.101 1/Å^3^ for metallic compounds, and 0.027 and 0.175 1/Å^3^ for covalent compounds. Once again, only metavalent solids reveal a smaller range of atomic densities spanning from 0.028 to 0.041 1/Å^3^.

The situation is different for the four remaining properties [ECoN, log(σ), Z*_+_, and *E*_G_], whose distributions show significantly stronger differences and thus a higher discriminatory power. This is obvious for the Born effective charge, which is 0 for metals, since the (nearly) free electrons of the metal screen the dipoles that could be created upon an atomic vibration. The metavalent compounds, on the contrary, have very high values of the excess Born effective charge Z*_+_, with values ranging from 1.7 to 3.3. The excess Born effective charge is less discriminative regarding ionic and covalent bonding, with values ranging from 0.6 to 1.4 for ionic compounds and 0.0 to 1.7 for covalent compounds. This also exemplifies the difficulties to separate ionic and covalent bonding based on material properties.

The room-temperature conductivity is a second quantity, where metals and metavalent solids are characterized by a well-defined range of (logarithmic) values that extends from 1.4 to 4 for the metavalent solids and from 3.3 to 5.8 for the metals. Again, the room-temperature conductivity is less discriminative regarding ionic and covalent bonding, with values ranging from −22.2 to −4.2 for ionic compounds and −14.0 to 3.0 for covalent compounds. Even the bandgap *E*_G_ is not well suited as a single criterion to distinguish ionic and covalent bonding, since both bonding mechanisms can lead to large bandgaps of 10.6 and 5.5 eV for ionic and covalent solids, respectively. However, small bandgaps are more frequently found in covalent solids than ionic solids, with covalent materials featuring bandgaps just above 0 eV, while the lower limit for ionic solids is 1.6 eV. Last, the ECoN not only is the largest for metals but also spans a wide range from 6.0 to 11.8 for this bonding mechanism. Both MVB and covalent bonding are characterized by narrow ranges, with values from 4.8 to 6.0 for metavalent solids and 3.1 to 6.0 for covalent solids. Ionic bonds span a wider range from 4.0 to 12.0. This discussion and the accompanying Fig. 3 show that there is no single property that can easily differentiate the different bonding mechanisms. Instead, Fig. 2 and Table 2 show that a classification based on several properties is more reliable, since the combination of several properties facilitates the clustering. Therefore, there could be interesting property correlations. One can look at such correlations in two dimensions, i.e., for two different parameters, an endeavor that will be undertaken next.

**Fig. 4.**
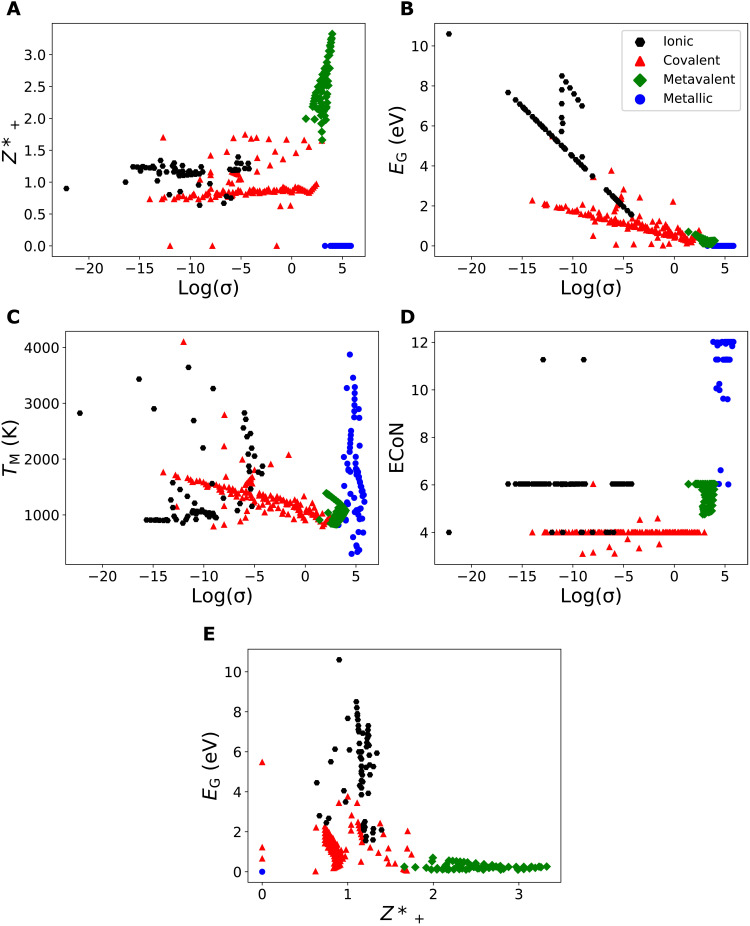
Correlation of different properties used for the clustering. (**A**) The logarithmic room-temperature conductivity plotted against the excess Born effective charge. (**B**) The logarithmic room-temperature conductivity plotted against the bandgap. (**C**) The logarithmic room-temperature conductivity plotted against the melting temperature. (**D**) The logarithmic room-temperature conductivity plotted against the ECoN. (**E**) The excess Born effective charge plotted against the bandgap. The colors and symbols indicate the bonding type of the corresponding compound: Black hexagons, ionic; red triangles, covalent; green diamonds, metavalent; blue circles, metallic (the expert classification was used).

Figure 4 shows some of these correlation plots for the different bonding mechanisms. Those correlations that are not shown in Fig. 4 are presented in fig. S1. It is quite instructive to discuss the correlation between the electrical conductivity at room temperature and the excess Born effective charge (Fig. 4A). For solids that use metallic bonding, the logarithmic room-temperature conductivity lies between 3.3 and 5.8. All these solids have a zero excess Born effective charge. Metavalent solids show very high values of Z*_+_, which even increases with increasing room-temperature conductivity. These data indicate that there has to be an interesting transition between increasing values of Z*_+_ with increasing conductivity for the metavalent solids and vanishing Z*_+_ for the metallic solids. This discontinuity is clear evidence for the different nature of metavalent and metallic solids. The data for the ionic and covalent solids are less informative, since they scatter around 1, with slightly higher average values for the ionic compounds.

In Fig. 4B, the correlation between the electrical conductivity at room temperature and the bandgap is depicted. All metals have a vanishing bandgap, independent of the electrical conductivity. For the metavalent solids, however, the bandgap increases with decreasing conductivity. This trend also continues for the ionic and covalent compounds, which have even lower conductivities. The data for ionic and covalent compounds seem to lie on two different lines. Ionic compounds have a larger bandgap for the same electrical conductivity.

In Fig. 4C, the correlation between the electrical conductivity at room temperature and the melting temperature is depicted. These data are interesting since the correlation differs significantly from the data in Fig. 4B. Now, the metals have a melting temperature that varies by almost an order of magnitude but is independent of the electrical conductivity. However, for covalent solids, the melting temperature shows a linear increase with a decrease in the logarithmic electrical conductivity. There is much more scatter for the ionic compounds, although a similar trend exists on average. For metavalent compounds, the melting temperature seems to be independent of the room-temperature conductivity.

In Fig. 4D, the correlation between the electrical conductivity σ at room temperature and the ECoN is displayed. Metals are characterized by high conductivities and high values of ECoN, yet there is no simple relationship between σ and ECoN. The same holds for the metavalently bonded systems, which have, on average, slightly lower σ and ECoN values. Again, no simple relationship between σ and ECoN is discernible. The same holds for covalent and ionic solids, which both show a narrow range of ECoN values but no simple correlation between σ and ECoN.

In Fig. 4E, the correlation between the bandgap and the excess Born effective charge is depicted. This figure can almost be seen as a consistency check. In Fig. 4A, the relationship between the electrical conductivity and the excess Born effective charge is depicted, while in Fig. 4B, the relationship between the electrical conductivity and the bandgap is displayed. It can hence be expected that there also will be a relation between the bandgap and the excess Born effective charge by transitivity. This is indeed the case. For metals, both the bandgap and the excess Born effective charge are zero. For the metavalent compounds, the Born effective charge, on average, decreases with increasing bandgap, but there is significant scatter. For ionic and covalent compounds, however, there is no clear tendency for the Born excess charge as a function of bandgap.

**Fig. 5.**
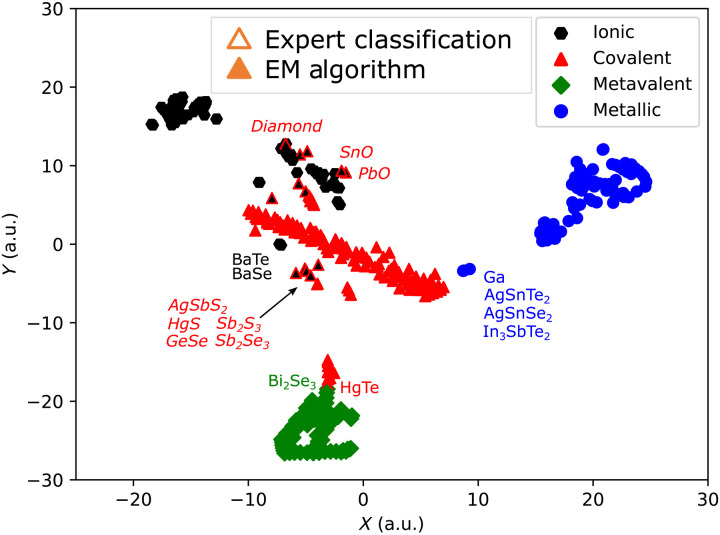
*t*-distributed stochastic neighbor embedding. The data points are positioned in a 2D chart, maintaining the (relative) distances of the 7D property space as good as possible. Labels in roman denote compounds close to a clusters of another bonding type, while labels in italic font correspond to misclassified compounds. A good separation of bonding types is achieved. Both metallic bonding and MVB form well-defined clusters. The distinction between ionic and covalent bonding, on the contrary, seems more challenging. a.u., arbitrary units.

The 2D plots presented here have revealed some interesting property correlations, which help to understand why the clustering based on material properties works well. However, one could ponder whether in *n*-dimensional space, the correlations will be even more insightful. To visualize these high-dimensional clustering results, a nonlinear low-dimensional embedding technique [*t*-distributed stochastic neighbor embedding (t-SNE)] is applied. This technique positions the data samples in a 2D chart while capturing the local structure of the data and revealing some global structure such as clusters. To do so, t-SNE describes the similarity between data points using joint probabilities. It then minimizes the Kullback-Leibler divergence between the joint probabilities of the high-dimensional data and the low-dimensional embedding. This leads to an intuitive grouping of the samples, i.e., compounds (see Fig. 5). It also shows the relative intercluster distances and the intracluster variance. However, the *x* and *y* axes of the 2D chart do not correspond to any of the seven individual physical properties in the dataset or simple combinations of them. Instead, they represent abstract parameters. Hence, the only meaningful information in the chart is the scalar-valued distances between the different materials. Figure 5 shows the t-SNE plot of all compounds in the database. While the labeling for four clusters is applied, note that the t-SNE distribution is inherently oblivious to the clustering results, i.e., the position of a material in this map is independent of its coloring.

The t-SNE shows that the clusters corresponding to different property portfolios and thus different bonding types are relatively well separated. The metallic cluster is rather isolated from the others, which is to be expected as metals have several rather distinct features, including an excess Born effective charge Z*_+_ and a bandgap *E*_G_ of zero, as well as high electrical conductivities at room temperature. The metavalent cluster is also rather well separated. This could be due to the large excess Born effective charge Z*_+_ in conjunction with well-defined values of ECoN and the room-temperature conductivity. There is a group of covalent materials that seem closely related yet discernible from metavalent compounds. Both groups have narrow bandgaps and moderate room-temperature conductivities. However, the metavalent compounds are octahedral-like, while the adjacent covalent compounds are tetrahedrally coordinated. The overall covalent cluster is the most fractured one. In the center, most of the covalent materials are located, and they are all classified correctly. This main cluster is surrounded by two subgroups, in which the classification by the two approaches partly disagrees. This finding reflects the complexity to distinguish between ionic and covalent bonding in compounds.

To provide a short interim summary, the success in classifying the properties of a large number of solids shows that there must be significant structure in the multidimensional property space. The different classes identified on the basis of material properties can be closely related to different bonding mechanisms. Therefore, the different chemical bonding mechanisms appear to be closely related to characteristic material properties. This conclusion is in line with the common belief that chemical bonding and material properties are related, yet it extends and quantifies these correlations. Furthermore, the close relationship between properties and bonding mechanisms has also been used to garner further evidence for MVB as a unique bonding mechanism distinct from metallic, ionic, and covalent bonding.

### From property space classification to material design

The close relationship between material properties and chemical bonding can also be exploited to design materials. The t-SNE plot in Fig. 5 provides a map-like representation of property space in 2D.However, the 2D coordinates of the embedding are artificial and hold (in themselves) no physical meaning. Thus, this map does not provide a recipe on how to navigate between positions in the t-SNE, e.g., to tailor the property portfolio of materials to optimize them for a given application.

**Fig. 6.**
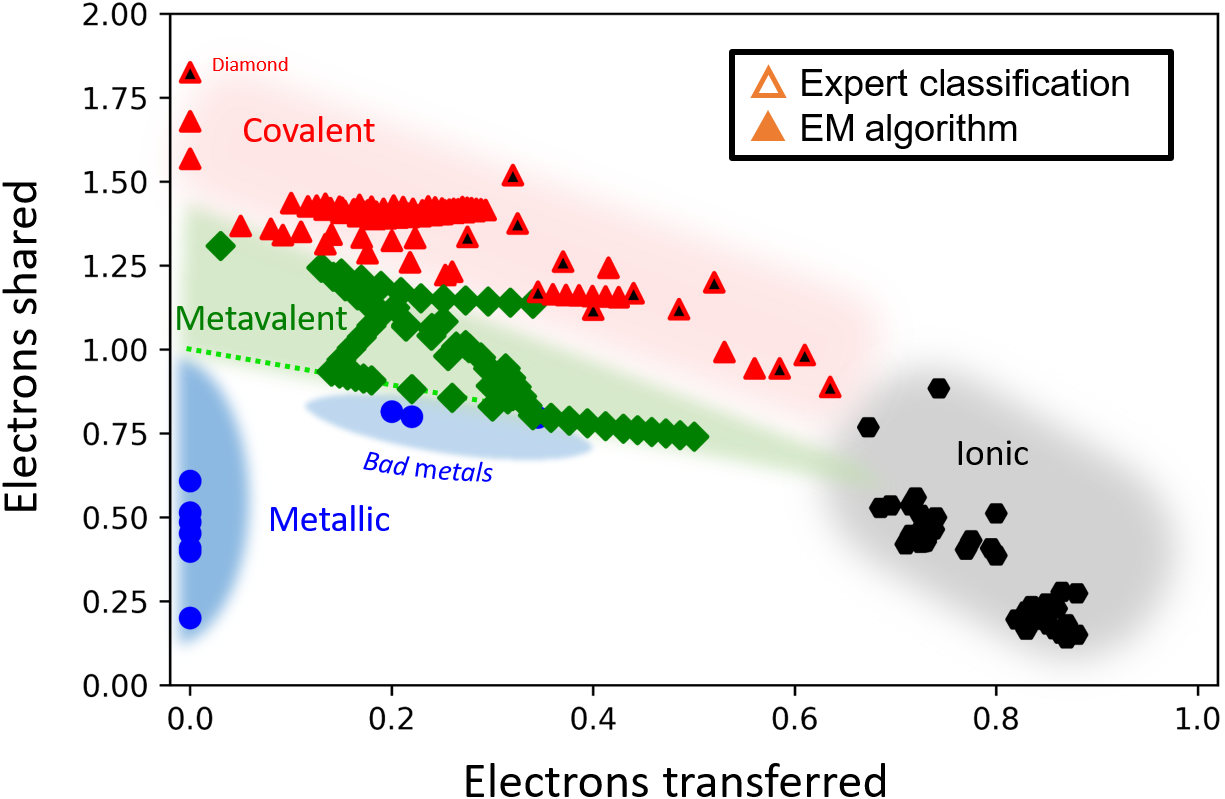
2D map classifying chemical bonding in solids. The map is spanned by the number of ES between adjacent atoms and the electron transfer renormalized by the formal oxidation state. Four different bonding mechanisms can be distinguished on the basis of their unique properties. Metallic (blue circles), covalent (red triangles), ionic (black hexagons), and metavalent (green diamonds) mechanisms of bonding are well separated, both using the EM algorithm and a classification based on scientific insights. The dashed line denotes the expected boundary between metavalent and metallic solids.

Chemical bonding, however, can be quantified by quantum chemical bonding descriptors. In particular, two quantities have been shown to quantify trends in bonding, the number of “ES” and “ET” (*16*, *23*). These quantities are derived from quantum-mechanical calculations based on the atomic arrangement of each compound. Using DFT and the QTAIM (*15*, *29*–*36*), one can determine how many electrons are shared (ES) between adjacent atoms or, more precisely, how many electron pairs can be formed between these atoms and how many electrons are transferred (ET) between atoms (*37*–*44*). This electron transfer is normalized by the formal oxidation state. Hence, an ET value of 1 for NaCl would indicate that one electron is transferred between Na and Cl, while the same ET value of 1 for MgO would imply that two electrons are transferred for MgO. An ES value of 2 refers to two electrons (or more precisely, one electron pair) being shared between adjacent atoms. High ET values in conjunction with low ES values are hence found for ionic compounds, while the opposite, low ET and ES values approaching 2 are characteristic for ordinary covalent materials (2 center–2 electron bonding, i.e., 2c–2e bonding). Metavalently bonded compounds are characterized by sharing only one electron, i.e., half an electron pair between adjacent atoms (ES around 1), while the ET values are small. Metals feature low ES and ET values, as their bonding is based on delocalized electrons. Figure 6 shows a plot of our database according to their ES and ET values. The colors and symbols indicate the bonding type classification derived here.

In this figure, we have included all compounds for which a quantum chemical bonding analysis has been performed and produced unambiguous results. It is interesting to compare Figs. 5 and 6. Both figures show a (clear) separation into four different classes, which apparently correspond to four different bonding mechanisms, i.e., ionic, covalent, metallic, and metavalent bonding. Hence, these two figures indeed provide further evidence for MVB as a distinct bonding mechanism besides ionic, metallic, and covalent bonding. Figure 6 also reveals that the borders between the different bonding mechanisms are quite well defined. This indicates that differences in chemical bonding, as described by different values of ES and ET, are also accompanied by clear differences in material properties.

**Fig. 7.**
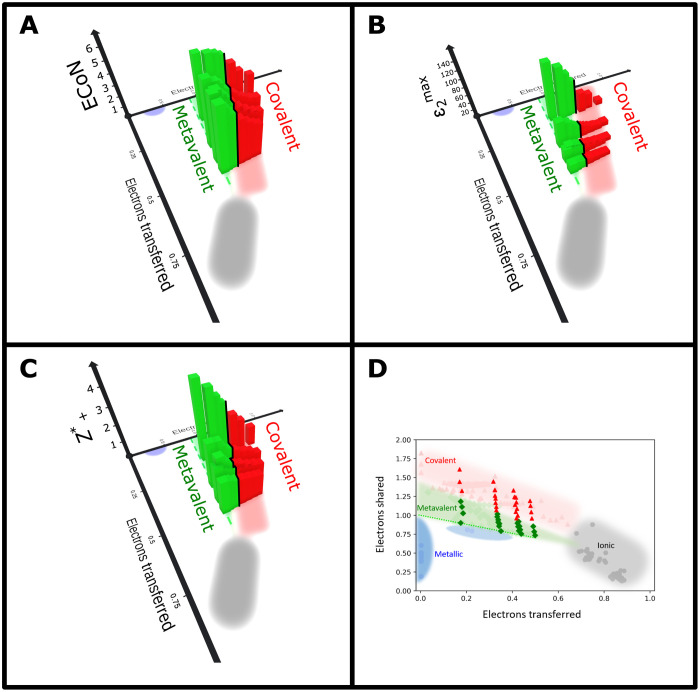
Various properties plotted against ES and ET for different monochalcogenides. The (stable) cubic systems are located along the green dashed line (ECoN of about 6). With increasing distortion, ECoN decreases, while ES increases. (**A**) ECoN, (**B**) maximum of the imaginary part of the dielectric function ε_2_(ω)_max_, (**C**) elevated Born effective charge Z*_+_, and (**D**) 2D version of the ES/ET map with the monochalcogenides highlighted.

While the connection between chemical bonding and material properties is established in chemistry and physics alike, Fig. 6 has shown that ES and ET are valuable, quantitative bonding descriptors. Hence, the question can be addressed if ES and ET are also good property predictors. This question can be answered if a given material property is depicted in a 3D figure as a height field function of ES and ET. If such a figure shows clear systematic trends of a material property as a function of ES and ET, then these two quantities are apparently good property predictors. Three relevant material properties are depicted as a function of ES and ET in Fig. 7, focusing on monochalcogenides, i.e., solids such as PbTe, PbSe, PbS, SnTe, SnSe, and SnS as well as GeTe and GeSe. Monochalcogenides play a prominent role as phase change materials (*27*, *45*), thermoelectrics, and topological insulators (*46*, *47*), i.e., they have interesting material properties. Therefore, it is potentially insightful to explore systematic property trends upon changing the quantum chemical bonding descriptors (ES and ET) for these materials. Figure 7A shows the dependence of the ECoN on ES and ET. Octahedrally coordinated solids (ECoN ≈ 6) with the property portfolio of metavalently bonded materials are all located on the dashed line in Fig. 6. This line is defined by the following equation

ES=1−0.52∙ET

Moving away from this line by increasing ES for constant ET decreases the ECoN, as shown clearly in Fig. 7.

Increasing ES for metavalent solids increases the Peierls distortion, which leads to a concomitant increase in the bandgap, as well as a decrease in electrical conductivity. These trends for metavalent solids can be explained by the unique bonding situation in metavalent solids, which are held together by a σ bond of p electrons, where for ET = 0, only half an electron pair (one electron) is located between adjacent atoms to realize a perfect octahedral arrangement (PD = 1).

Figure 7B shows systematic changes for the maximum photon absorption, i.e., ε_2_(ω)_max_ for these monochalcogenides as a function of ES and ET. This figure reveals a remarkably clear trend for ε_2_(ω)_max_. This quantity decreases with increasing ET and ES increasing away from the green dashed line shown in Fig. 6, i.e., upon increasing Peierls distortion. These clear trends can be explained thoroughly. The strength of the optical absorption, i.e., ε_2_(ω)_max_, in these monochalcogenides depends, according to Fermi’s golden rule, on the joint density of states and the matrix element for the optical transition. The trends seen in Fig. 7B are governed by changes of the matrix element for the optical transition, which is dominated by the overlap of the wave functions for the initial and the final state. Increasing ET decreases this overlap, which also decreases upon increasing ES away from the green dashed line in Fig. 6. Hence, this explains the decrease in the overlap and hence ε_2_(ω)_max_ (*48*, *49*) upon increasing ET and increasing ES (above the green dashed line).

Figure 7C lastly shows systematic changes for the chemical bond polarizability, i.e., the elevated Born effective charge Z*_+_. Again, this figure reveals clear trends. Z*_+_ decreases, on average, with increasing ET along the green dashed line and decreases systematically upon increasing ES away from this line. Comparing Fig. 7 (A and C) with Fig. 4 (A and D) shows that the trends for both ECoN and Z*_+_, i.e., trends in atomic arrangement (size of the Peierls distortion), are more clearly discernible if ECoN and Z*_+_ are depicted as a function of ES and ET. This consolidates the conclusion that ES and ET are excellent property predictors for the chalcogenides discussed here. This implies that there is stronger coherence in the material properties if we chose ES and ET, as a “natural” parametrization to describe systematic trends for the properties of metavalent solids, instead of a property descriptor like the electrical conductivity.

Figure 7B reveals why this is the case. The electrical conductivity of metavalent solids decreases with increasing ES, i.e., if one moves away from the green dashed line. However, it also decreases with increasing ET on the green dashed line. In both cases, this leads to an increase in the electron localization. Nevertheless, only an increase in ES reduces the ECoN. Hence, ES and ET, but not σ, are ideally suited to describe trends for a number of physical properties including *E*_G_, Z*+, ECoN, σ, and ε_2_(ω)_max_.

It can be seen that the metavalent materials with the strongest absorption are found for a given ET at the smallest ES value compatible with MVB, i.e., the dashed line in Fig. 7. This is apparently the limiting line for MVB. For an octahedral system with fewer bonding electrons, i.e., smaller ES, the Fermi energy *E*_F_ moves into the valence band, i.e., turns into a metal. Will this be accompanied by property changes? For this particular border, we expect the most pronounced effects for the Born effective charge, while the changes for σ and *E*_G_ are apparently much less pronounced. This is supported by Fig. 4A, which shows that in metals, the Born effective charge is zero, yet in metavalent solids, the Born effective charge increases significantly when the electrical conductivity increases and approaches the values found in bad metals, i.e., log(σ) ≈ 4. Hence, a very interesting and pronounced transition of Z*_+_ at the border between MVB and metallic bonding is expected (green dashed line in Fig. 7). The map in Fig. 6 helps to identify materials that are potential end members of a pseudo-binary line that crosses the border from MVB to metallic bonding. One such system is AgSbTe_2_-AgSnTe_2_. We are presently studying the nature of the property changes for this pseudo-binary line. Another system with unconventional metallic properties is In_3_SbTe_2_, which has a metallic state in the crystalline phase just below the green dashed line in Fig. 6 (see also Table 2) yet is covalently bonded and has a bandgap in its amorphous state (*50*).

In summary, it has been shown that the property space consisting of the electrical conductivity σ, Born effective charge Z*, ECoN, bandgap *E*_G_, melting point *T*_M_, density ρ, and atomic density ρ_A_ is intrinsically structured. Hence, using the Gaussian mixture helps to classify about 330 materials. This computational approach only uses uncontroversial material properties as input and is therefore unbiased in terms of chemical or physical assumptions and conventions regarding bonding. The resulting clustering is most discriminative for four different classes. The different classes can be attributed to different bonding mechanisms, i.e., covalent, metallic, and ionic bonding as well as MVB. This algorithm is only challenged if covalent and ionic bonding have to be distinguished, which can be attributed to the continuous transition between these two bonding types. The distinct property changes upon the transition from MVB to covalent bonding, as well as from MVB to metallic bonding, support the view that MVB is a distinct bonding mechanism besides ionic, metallic, and covalent bonding. MVB has already been identified as a unique bonding mechanism in another recent publication. It revealed that MVB is characterized by unique quantum-level descriptors derived from a Hirshfeld analysis, which can help to identify solids utilizing MVB (*26*). Here, we focus on property space instead, which is crucial to tailor advanced functional materials.

The conclusion that property space has considerable structure and that this structure is related to differences in chemical bonding provides an alternative pathway to materials design. ES and ET have already been shown to be good quantum chemical bonding descriptors. However, they are also good property predictors as demonstrated here. They provide natural variables to describe systematic property trends, in particular, for metavalent solids. Hence, they can be used to tailor application-relevant properties. Recently, systematic trends for the switching kinetics of phase change materials (*51*) or the performance of thermoelectrics have been attributed to systematic changes of bonding in metavalent solids (*25*). Even the bond rupture in atom probe tomography has provided further evidence for the unconventional bonding mechanism in this material class (*52*).

The relationship between material properties and chemical bonding mechanisms indicates that we can use the suite of methods of machine learning and classification to look for materials with a specific combination of properties and not only explain and tailor a singular property of a given compound. With this approach, it might even be possible to phrase general statements whether certain property portfolios can even exist or whether the attempt to create such a material is bound to fail.

Two important steps will extend the value of this approach further, increasing the database of materials and material properties to work with and extending this concept to more complex solids. Having a larger material and property base will help to substantiate the claims of the predictive power of the concept introduced. Extending this approach to solids where crucial material properties depend on several different bonds will help verify whether certain material properties can really be attributed to single bonds in a complex solid. This could recently be achieved for halide perovskites, where the strong optical absorption in the visible range and the small effective masses could be attributed to the unique bond between the halogen and group IV (Sn/Pb) atom (*24*). Hence, it seems very plausible that the framework for material design outlined in Fig. 1 can be successfully used also for more complex solids, where material properties are governed by different bonds.

## MATERIALS AND METHODS

### Data acquisition

About 130 unique materials were used, mainly taken from (*16*). Properties and additional materials were compiled using openly available databases (*13*, *14*) and/or computed. The density of data points was augmented by blending materials and interpolating the properties, if the corresponding phase diagram allowed for it, increasing the total number of data points to approximately 330 compounds. A complete list of all compounds and their properties is included in the Supplementary Materials.

### Classification algorithm

The dataset of about 330 compounds is evaluated by a variant of the EM algorithm (*18*) to fit a GMM. The number of modes is varied to set the number of formed clusters. We did not use the option to exploit constraints on the covariance matrix in our GMM, since we wanted to make sure that the numerical clustering algorithm is as unbiased as possible. We assume that the distribution of the materials in property space can be approximated by a GMM, but we do not speculate a priori about the particular relations and dependencies of individual material properties.

### Nonlinear low-dimensional embedding technique (t-SNE)

t-SNE compares the similarity between data points using joint probabilities. The Kullback-Leibler divergence between the joint probabilities of the high-dimensional data and the low-dimensional embedding is then minimized, resulting in an intuitive grouping of the data points.

### Computational details

DFT using projector augmented wave potentials (*53*) was used to calculate further compounds and their properties. Perdew–Burke-Ernzerhof (PBE) functionals were used (*54*). The software implementations were provided by ABINIT (*55*, *56*) and Quantum Espresso (*57*, *58*). The energy cutoffs exceeded 500 eV for all computations.

The DGRID (*59*) and Critic2 (*40*) codes were used to calculate the ES and ET values using Bader basins (*60*). The integration over the electron density of a single basin yields the electron population of the respective atom. By subtracting the nominal charge of said atom, the total number of ET (TET) is obtained. Dividing TET by the formal oxidation state, the relative number of ET is calculated. The number of ES, in contrast, is calculated by integrating over the exchange-correlation hole over the basins of the two atoms of interest.
