# Supplementary material for: Classification of properties and their relation to chemical bonding: Essential steps toward the inverse design of functional materials
Source: Sci Adv. 2022 Nov 25;8(47):eade0828. doi: 10.1126/sciadv.ade0828 (PMC9699668; doi:10.1126/sciadv.ade0828)
Supplement: Supplementary file 1 — Supplementary Text Tables S1 and S2 Figs. S1 to S3 [file sciadv.ade0828_sm.pdf]

Science Advances
AAAS

Supplementary Materials for

Classification of properties and their relation to chemical bonding: Essential steps toward the inverse design of functional materials

Carl-Friedrich Schön *et al.*

Corresponding author: Matthias Wuttig, wuttig@physik.rwth-aachen.de

Sci. Adv. **8**, eade0828 (2022)
DOI: 10.1126/sciadv.ade0828

The PDF file includes:

Supplementary Text
Tables S1 and S2
Figs. S1 to S3
Legend for data S1

Other Supplementary Materials for this manuscript includes the following:

Data S1

I. The Expert Classification

II. Property correlations

III. Classification metrics

IV. Evolution of cluster formation

V. Using the quantum-chemical bonding map to design and predict properties

VI. Full dataset of materials and properties in Data S1 (separate file)

The supplementary information summarizes crucial information about the methods employed to derive the data presented and conclusions drawn. The expert classification describes the criteria and property limits employed to classify different bonding mechanisms. Subsequently, two-dimensional property correlations are discussed. Furthermore, the quality of the classification is evaluated. Then, the evolution of the cluster formation is described and finally the concept developed is utilized to predict and design properties.

I. The Expert Classification:

The classical assignment of bonding types to each material is rather simple and unequivocal for metals, which are defined by a vanishing bandgap E_G= 0 eV, (leading e.g. to high electrical conductivity at room temperature). For the other bonding types, no such absolute criterion exists. Ionic and covalent bonding describe two opposing concepts on how chemical bonds can be achieved in a solid. For ionic bonding, electrons are transferred from one atom to another, creating a negatively charged anion and a positively charged cation with a net charge difference. The electrostatic interaction between these ions then produces an attractive force which leads to bonding. For a covalent bond to form, electron pairs are formed between atoms, i.e. electrons are shared. This configuration lowers the total energy of the system and stabilizes the atomic configuration (It should be noted that only material properties were used for the classification, and not values of Electrons Transferred and Electrons Shared). While for an ionic bond two different elements constitute the bonding atoms (e.g. Na and Cl), a (pure) covalent bond can only be achieved by two identical elements (e.g. in diamond, consisting only of carbon atoms). However, for all non-monoatomic compounds, a certain amount of electrons is always transferred, while some are always shared. Hence covalent and ionic bonding contributions are always present to a varying degree, leaving some compounds to be ambiguous to which type they ultimately belong. These compounds are sometimes labeled as "ionocovalent" (e.g. ZnO). Metavalent bonding is characterized by low to moderate charge transfer in combination with an approximately half-filled p-band. While such a half-filled band would normally lead to a vanishing bandgap E_G= 0, small distortions in the structure and/or the charge transfer break the symmetry of the system and open up a small bandgap. Metavalent compounds can therefore be thought of as "Incipient Metals" as well(19). This competition between localization and delocalization of the electrons creates a unique property portfolio of compounds utilizing MVB, including a high Born Effective Charge Z*, a high optical dielectrical constant ε_∞ and a high Grüneisen parameter γ_{TO}, which is a measure of anharmonicity (softness) of the compounds.
In a 'Gedanken'experiment another import criterion can be established. If there is a close relationship between distinct material properties and distinct bonding mechanisms, transitioning from one bonding mechanism to another should show discontinuous behavior of one or more properties. Such border transitions were investigated by Guarneri et al.(48), showing that discontinuous behavior indeed does exist between covalent and metavalent compounds. Unfortunately, such border transitions cannot be realized for all compounds and their existence for some systems does not strictly prove that they exist for all systems (and among all borders). The assignment of (and affection for) chemical bonding types by chemists and physicists might hence still be biased by engrained heuristic knowledge. By employing an a priori unbiased clustering algorithm, we will show that the concept of distinct bonding types holds true even for a purely data-driven approach.

Table S1 shows the minimum and maximum values of each property within the respective bonding types, as assigned traditionally by the expert classification (within the database utilized for the classification).

Table S1: ***Characteristic property ranges for ionic, covalent, metavalent and metallic bonding.***

	Ionic		*Covalent*		*Metavalent*		*Metallic*	
	Min	*Max*	*Min*	*Max*	*Min*	*Max*	*Min*	*Max*
Conductivity Log(σ)	-22.2	-4.2	-14.0	3.0	1.4	4.0	3.3	5.8
Elev. Born Eff. Charge Z^*_+	0.6	1.4	0.0	1.7	1.7	3.3	0.0	0.0
ECoN	4.0	12.0	3.1	6.0	4.8	6.0	6.0	12.0
Band Gap E_G (eV)	1.6	10.6	>0	5.5	0.1	0.7	0.0	0.0
Melting Point T_M (K)	853	3643	794	4100	828	1387	301	3873
Density ρ (g/cm^3)	1.9	5.8	2.3	8.6	5.9	7.9	0.6	21.9
Atomic Density ρ_A (10^{-2}/ Å^3)	1.7	16.8	2.7	17.5	2.8	4.1	1.4	10.1

It becomes apparent that individual properties are not sufficient to assign a bonding type, as the ranges do overlap. The only exceptions are metallic compounds, for which the elevated Born effective charge Z^*_+ and the band gap E_G are always 0.

II. Property Correlations:

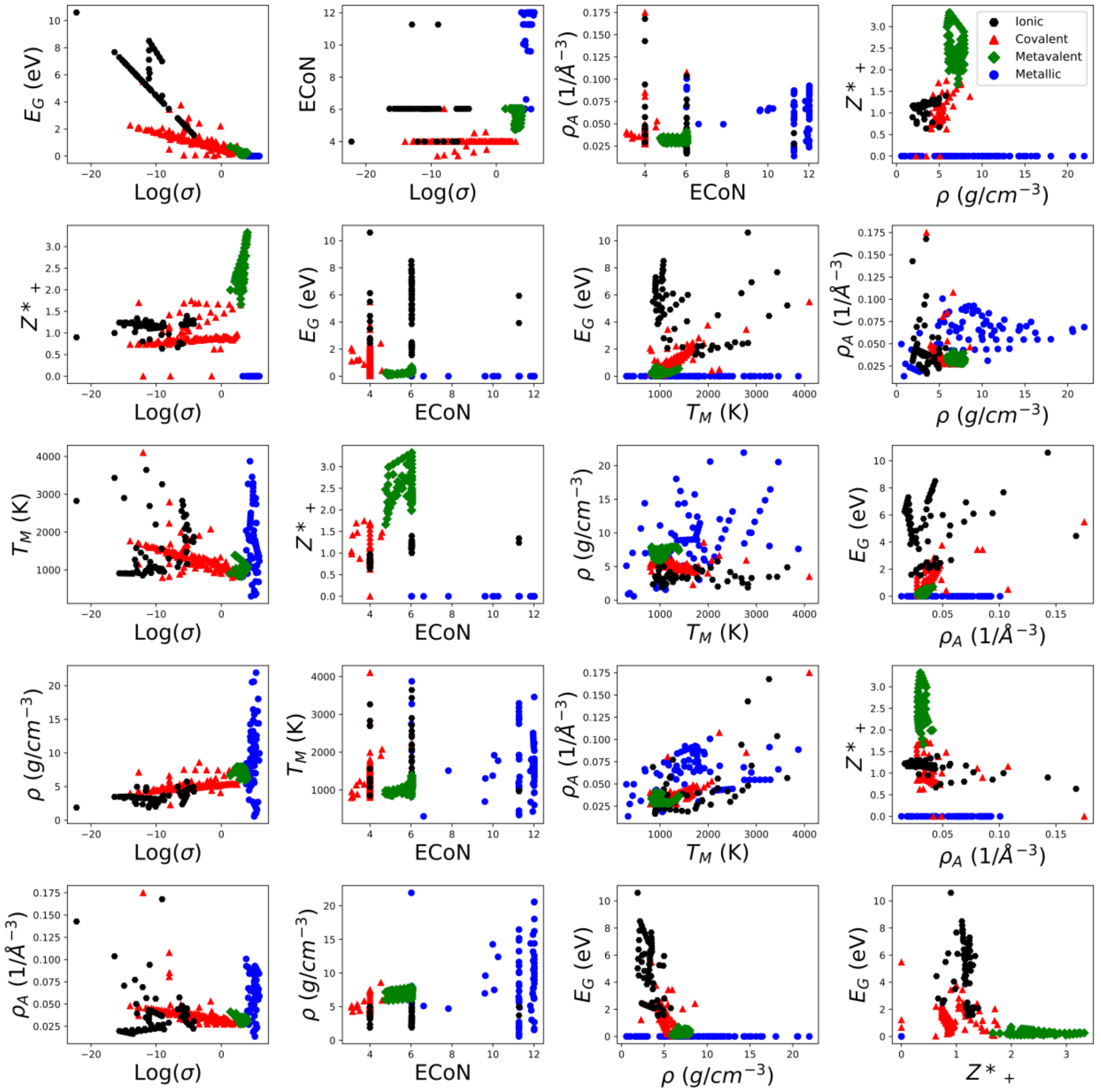

*Figure S1: **Correlation of different properties used for the clustering**. The colors indicate the bonding type of the corresponding compound: Black Hexagons: Ionic, Red Triangles: Covalent, Green Diamonds: Metavalent, Blue Circles: Metallic (The expert classification was used)*

Figure S1 shows the correlation of all properties with each other. While some of these plots shows pronounced correlations and novel insights, e.g. Z^*_+ or the band gap E_G vs. the electrical conductivity, for other property combinations no clear correlation is visible by eye. This shortcoming is particularly pronounced if a certain property range is not characteristic for a certain bonding mechanism, as found for e.g. the melting temperature, the atomic density or the mass density.

III. Classification Metrics:

Different metrics can be utilized to evaluate the classification result of the Expectation Maximization algorithm (EM algorithm).

Figure S2 shows the Average Log Likelihood (ALL) metric for different numbers of allowed clusters.

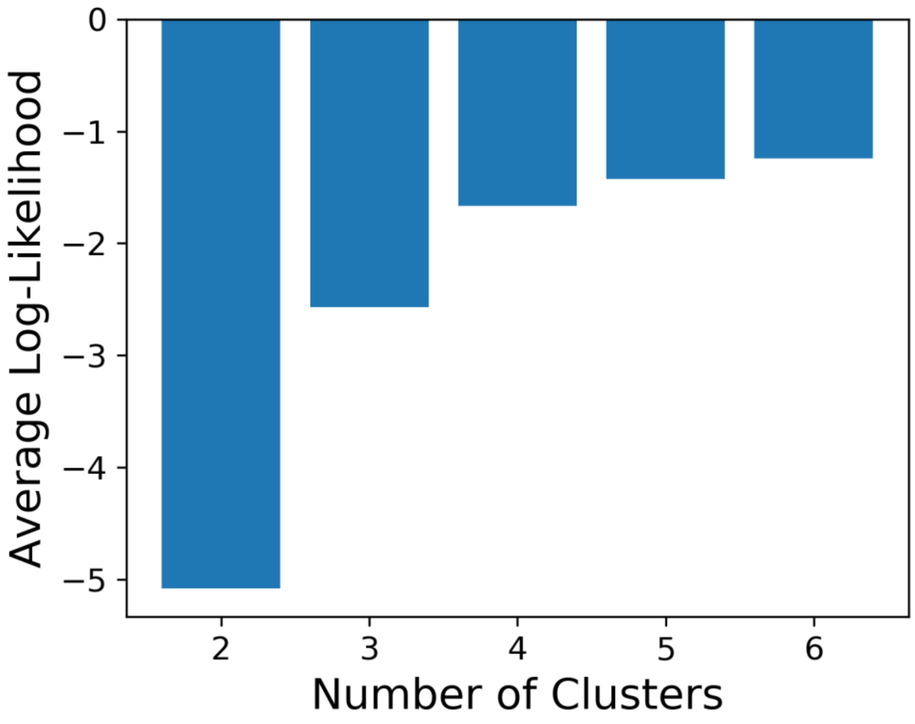

Figure S2: ***Average Log-Likelihood for 2-6 Clusters.*** *Higher values indicate better results.*

It can be observed that the classification quality increases monotonously with an increasing number of allowed clusters (higher values are better). This is expected, as adding an additional cluster is comparable to adding a degree of freedom, which is bound to improve the quality of any regression.

However, looking at the differences of the ALL values going from n to n+1 clusters, it becomes apparent that the change is quite large going from 2 to 3 and from 3 to 4 clusters, while it is less significant going from 4 to 5 and from 5 to 6. This indicates that the classification quality is improving substantially up to a number of 4 possible clusters.

IV. Evolution of Cluster Formation:

The EM algorithm analysis of the material properties is purely based on a measure for the overall clustering quality (intra-cluster coherence) but it provides only little insight into the relative similarity between clusters (inter-cluster correlation). In order to assess this aspect as well, the EM clustering is redone with increasing numbers of Gaussian modes and track how clusters split, i.e., how the samples of a coarse cluster are distributed into finer clusters.

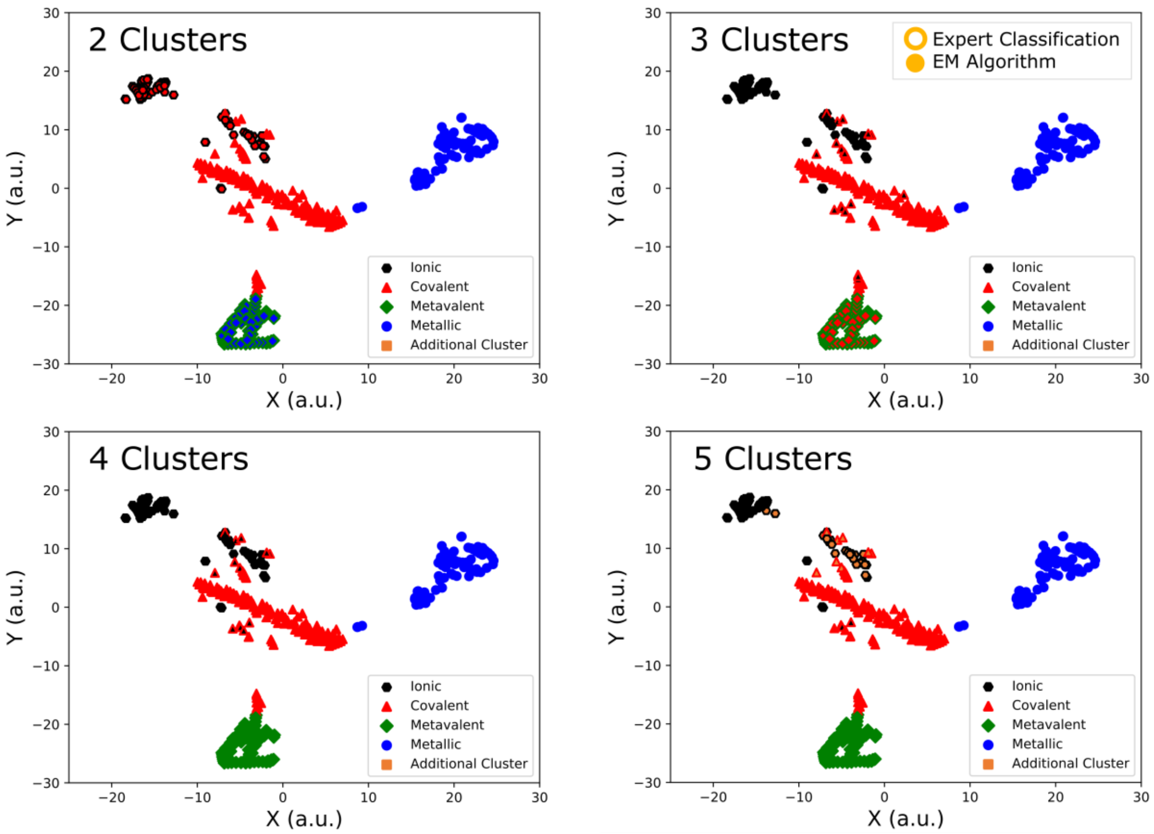

Figure S3: ***Sequence of clustering results for different numbers of clusters, utilizing the T-distributed stochastic neighbor embedding (t-SNE).*** *For 2 clusters, ionic and covalent, as well as metals and MVB materials are grouped together. For 3 clusters the metals are separated out, while the MVB materials switch to the covalent cluster. 4 clusters reproduce the expert classification to a large extent, while 5 clusters split off some covalent and ionic materials.*

Figure S3 shows that for 2 clusters, metals and MVB compounds form a cluster and are separated from a cluster of ionic and covalent materials. For 3 possible clusters, metals and ionic compounds are separated, while Covalent and MVB materials are clustered together. 4 Clusters have already been discussed, and for 5 clusters the covalent and ionic group split of in three clusters.

This shows that up until a number of three clusters, the traditional allocation of compounds according to chemistry is reproduced, with the MVB compounds being part of the covalent group. However, going to 4 clusters is not only numerically favorable (see figure S3), it flawlessly retrieves the materials which are assigned to be metavalent compounds. This notion of metavalently bonded systems being special is further underlined by the fact that the MVB materials were joined with the metals for two clusters, while changing to be joined with the

covalent cluster for three clusters, meaning they are equally different from covalent and metallic systems alike. Hence putting them together with covalent or metallic systems when forced to is indifferent for the algorithm.

V. Using the quantum-chemical bonding Map to predict and design properties

An advantage of the ES/ET based map (see figure 6) is that navigation within this framework is achieved much easier. For example, a possible way to increase/decrease the ET value of a compound is to replace one constituent with another element from the same group of the periodic table of elements. This way the number of valence electrons is preserved, but it changes the electronegativity, increasing or decreasing the electron transfer. This can be exemplified by looking at the position of PbTe, PbSe and PbS. While the ES values change from 0.8 to 0.76 and 0.74, respectively, the ET values change from about ET=0.34 (PbTe), over ET=0.43 (PbSe) to ET=0.5 (PbS).

While the density of data points in figure 6 is not yet sufficient to inversely design a material by picking a property set of choice, we can show a proof of principle example with reduced complexity. The compound ZnS in wurtzite structure was excluded from the general dataset, as initially no value for the Born Effective Charge Z* was available. ES, ET, conductivity σ and band gap E_G are known however, and a value for Z* was computed to complete the property set. We can therefore try to predict these properties of ZnS (Wurtzite) and compare it with the corresponding values from literature. In order to predict properties from ES/ET coordinates, the four closest compounds surrounding ZnS (Wurtzite) with higher/lower ES and ET respectively were chosen (ZnS, CdS, SnS, PbTe:0.2-Bi_2Te_3:0.8) and the values for conductivity σ and band gap E_G are calculated by employing bilinear interpolation. As however PbTe:0.2-Bi_2Te_3:0.8 employs metavalent bonding, and we expect a non-continuous property behavior crossing bonding borders, this point was excluded from the interpolation.

The results are summarized in table S2:

Table S2: ***Comparison between literature and predicted values for ZnS in wurtzite structure.***

ZnS (Wurtzite)<br>ET=0.42 / ES= 1.22	Log(σ)	E_G	Z^*_+
Prediction	-5.0	2.3 eV	1.23
Literature Value	-4.2	2.0 eV	1.0

The table shows that the predicted values are in reasonable agreement (about 15% difference) with the values from literature, underlining the validity of our approach.

VI. Full dataset of materials and properties in Data S1 (separate file)

All compounds with their respective classification results, properties and ES/ET values are listed in the separately attached CSV file: **Data S1.csv**.
